# Individual and contextual factors associated with under- and over-nutrition among school-aged children and adolescents in two Nigerian states: a multi-level analysis

**DOI:** 10.1017/S1368980022000258

**Published:** 2022-08

**Authors:** Adeleye Abiodun Adeomi, Adesegun Fatusi, Kerstin Klipstein-Grobusch

**Affiliations:** 1 Department of Community Health, College of Health Sciences, Obafemi Awolowo University, Ile-Ife, Osun, Nigeria; 2 Division of Epidemiology and Biostatistics, School of Public Health, Faculty of Health Sciences, University of the Witwatersrand, Johannesburg, South Africa; 3 Centre for Adolescent Health and Development, School of Public Health, University of Medical Sciences, Ondo, Nigeria; 4 Julius Global Health, Julius Center for Health Sciences and Primary Care, University Medical Center Utrecht, Utrecht University, The Netherlands

**Keywords:** Thinness, Overweight, Obesity, Double burden of malnutrition, Nutritional status, Contextual factors, Multi-level analysis

## Abstract

**Objective:**

This study aimed to identify individual and contextual factors that are associated with under- and over-nutrition among school-aged children and adolescents in two Nigerian states.

**Design:**

Community-based cross-sectional study.

**Setting:**

The study was carried out in rural and urban communities of Osun and Gombe States in Nigeria.

**Participants:**

A total of 1200 school-aged children and adolescents.

**Results:**

Multi-level analysis showed that the full models accounted for about 82 % and 39 % of the odds of thinness or overweight/obese across the communities, respectively. Household size (adjusted OR (aOR) 1·10; *P* = 0·001; 95 % CI (1·04, 1·16)) increased the odds, while the upper wealth index (aOR 0·43; *P* = 0·016; 95 % CI (0·22, 0·86)) decreased the odds of thinness. Age (aOR 0·86; *P* < 0·001; 95 % CI (1·26, 8·70)), exclusive breastfeeding (aOR 0·46; *P* = 0·010; 95 % CI (0·25, 0·83)), physical activity (aOR 0·55; *P* = 0·001; 95 % CI (0·39, 0·78)) and the upper wealth index (aOR 0·47; *P* = 0·018; 95 % CI (0·25, 0·88)) were inversely related with overweight/obesity, while residing in Osun State (aOR 3·32; *P* = 0·015; 95 % CI (1·26, 1·70)), female gender (aOR 1·73; *P* = 0·015; 95 % CI (1·11, 2·69)) and screen time > 2 h/d (aOR 2·33; *P* = 0·005; 95 % CI (1·29, 4·19)) were positively associated with overweight/obesity.

**Conclusions:**

The study shows that selected community and individual-level factors are strongly associated with thinness and overweight/obesity among school-aged children and adolescents.

Obesity is a global challenge, and the prevalence of overweight and obesity is increasing at a faster rate in low- and middle-income countries as compared with high-income countries^(1)^. At the same time, the challenge of under-nutrition persists still in many low- and middle-income countries^(2)^. Thus, many low and middle-income countries are confronted with a double burden of malnutrition^(3)^, with co-existing high level of under-nutrition and an increasing level of over-nutrition^(4)^. Several factors are associated with malnutrition, and the ecological systems model has been proposed to understand child nutrition processes^(5)^. Ecological systems are the contextual factors within which individuals are nested^(6,7)^.

While the ecological systems theory recognises the impact of the child’s individual factors in affecting development, it conceptualises that these factors are just one group out of many others^(6,8)^. In the original work on the ecological systems theory, four different inter-related environments are identified, with the microsystem as the most proximal level, followed by the mesosystem, the exosystem and the macrosystem level. While the most proximal level reflects individual and intrafamilial processes, the outermost system reflects the cultural, religious and socio-economic organisation of the community^(6,8)^. The chronosystem is the fifth and final level of Bronfenbrenner’s ecological systems theory; it encompasses the concept of time and consists of the environmental changes and transitions that occur over the lifetime and influence the child’s development. The theory was later updated to emphasise the important role the individual plays in the development process^(7)^. It was hypothesised that human development was the result of the interplay among four processes: person, context, process and time, with ‘person’ factors to mean individual characteristics, the ‘context’ to refer to the external factors described in the original work on the ecological systems theory, the ‘process’ to refer to the interaction between the person and the context and this development to be understood with reference to ‘time’^(7,8)^.

Most of the research efforts targeted at identifying the determinants of the nutritional status of school-aged children and adolescents have focussed mainly at the individual factors alone. A number of studies have reported a significant relationship between nutritional status and such factors as age and gender of the child^(9,10)^, residence^(11,12)^, physical activity^(13,14)^ and feeding patterns^(11,15)^ of children. However, there is little evidence on the relationship between the nutritional status of school-aged children and adolescents and the communities within which the children live. Little or no evidence exists in Nigeria about the relationship between the nutritional status of this group of children and community-level factors, and very few studies have explored ecological factors as determinants of child nutrition beyond the individual factors.

There is a need to focus on the determinants of the nutritional status of school-aged children and adolescents in Nigeria, because identifying the determinants has not been the focus of most research efforts on the subject in Nigeria^(16–18)^. The interest of majority of the researchers in this field in Nigeria has been the assessment and description of the nutritional status. Identifying the determinants is important, not only in improving the understanding about the subject, but especially in planning appropriate nutritional interventions for the children.

Identifying the individual and contextual determinants, using multi-level modelling however, has added advantages. Firstly, conventional regression models assume the units of analysis are not dependent, and this is not usually true. This error may then lead to over-estimation of statistical significance^(19)^. Designing interventions based on erroneous evidence may lead to ineffectiveness of such interventions. Additionally, identifying the contextual determinants will help to understand the dynamics of the influence of the contextual units^(19)^, and especially the potentials they hold for reducing the burden of a complex health challenge such as the double burden of under- and over-nutrition.

While multi-level modelling has been used to explain the determinants of the nutritional status of children under the age of 5 years in Nigeria^(20,21)^, to date, data are lacking for school-aged children and adolescents in Nigeria. This study, therefore, aimed to identify individual and contextual factors that are associated with either under- or over-nutrition among both school-aged children and adolescents in two Nigerian states.

## Materials and methods

### Study design and setting

This community-based cross-sectional study was carried out in two Nigerian states. School-aged children and adolescents aged between 6 and 19 years and their mothers formed the study population. The sample size was calculated to get an absolute precision of ± 5 % for prevalence estimates using STATCALC on the Epi-Info software^(22)^, and a design effect of 1·5, which is an adjustment for the sampling technique (multi-stage) used. After correcting for an anticipated non-response rate of 10 %, the sample size came to 561 and was rounded off to 600 for each state, making a total of 1200.

The classification of Nigeria’s six geo-political zones in Nigeria according to their wealth index by the Nigeria demographic and health survey^(23)^ was used in selecting the states for this study. One state each from the zones with the lowest (North-east zone) and the highest wealth index (South-west zone) were selected using simple random sampling technique (balloting method). Selection of states was done according to wealth index and geo-political zones because these were the two most consistent factors that were significantly associated with malnutrition from previous similar studies in Nigeria^(20,21,24)^. Gombe and Osun States were selected from the North-east and the South-west zones, respectively. The 1200 children 6–19 years old and their mothers who constituted the study population were then selected using the multi-stage sampling technique.

### Data collection

Data collection was carried out with interviewer-administered pre-tested structured questionnaires using REDCap^(25)^, a data collection software installed on electronic tablets. The mothers were the respondents for the sections on general characteristics of the child, household/family characteristics and community factors. The school-aged children or adolescents responded to the sections on the dietary diversity, physical activity patterns and pubertal staging. Anthropometric measurements of the children and mothers were taken according to standard protocols recommended by the International Society for the Advancement of Kinanthropometry^(26)^. Weight was measured in 0·1 kg by use of Omron® electronic bathroom weighing scale. Height was measured to the nearest 0·1 metre using a stadiometer. Weighing scales were routinely standardised by the use of known weights.

Screen time was the time in hours that the child spent with television, computer, video games or phones/d. The physical activity was assessed using the physical activity questionnaire for older children and adolescents by Kowalski *et al*.^(27)^ from which a composite score of 1 to 5 was derived (higher scores represent higher physical activity levels).

### Statistical analyses

#### Measures

The primary outcome/dependent variable was the nutritional status, which was assessed using the BMI-for-age according to WHO reference values^(28)^. It was categorised into: (1) thinness; (2) normal and (3) overweight/obese. The independent variables used were those that had been previously reported from similar studies^(20,21)^ and are presented in Table 1. The independent variables consist of three groups of variables: individual, household and community-level factors, but the individual and household-level factors were collapsed into the individual-level factors for the multi-level analysis. Communities were taken as those who shared a common enumeration area, which served as the primary sampling unit. Household wealth index was calculated using ownership of some household possessions, as it was used by the Nigeria demographic and health survey^(23)^. Principal component analysis was then used to produce a common factor score (household wealth index score) for each household. These scores were used to categorise wealth index into three: (1) poor; (2) middle and (3) rich. The community wealth index was calculated by finding the median wealth index score for each community, and these were then categorised into: (1) low and (2) high using the median value. Pubertal staging was assessed using the Tanner pubertal self-rating scale^(29)^.

**Table 1:** Definitions for independent variables

Independent variables	Description
Individual-level factors	
Age of child (years)	Expressed as a continuous variable
Sex of child	Categorised into (1) male (2) female
Birth order	Categorised into (1) 1 (2) 2–4 (3) > 4
Education	Categorised into (1) currently in school (2) previously in school (3) never in school
Birth weight	Categorised into (1) low < 2·5 kg (2) normal 2·5–4 kg (3) > 4 kg
Breastfeeding duration	Expressed as a continuous variable (months)
Immunisation status	Categorised into (1) complete (2) incomplete
Birth place	Categorised into (1) others (2) hospital
Exclusively breastfed for 6 months	Categorised into (1) yes (2) no
Child’s health	This is the mother’s perception of the child’s health and it was categorised into (1) very good, (2) good and (3) not too good
Puberty stage	Categorised into stages 1–5 (Tanner staging)
Screen time (in hours)	Categorised as (1) < 2 h (2) ≥ 2 h
Physical activity	Expressed as continuous variable (scores)
Household/family factors	
Household size	Expressed as continuous variable
Number of children	Expressed as continuous variable
Wealth index	Categorised into (1) Poor, (2) Middle and (3) Rich
Marital status	Categorised into (1) single, (2) married and (3) previously married
Family type	Categorised into (1) monogamous and (2) polygamous
Maternal education	Categorised into (1) less than secondary and (2) secondary or more
Father’s education	Categorised into (1) less than secondary and (2) secondary or more
Mother’s unemployment	Categorised into (1) employed and (2) unemployed
Community-level factors	
State	Categorised into (1) Gombe and (2) Osun
Residence	Categorised into (1) rural and (2) urban
Community wealth index	Categorised into (1) low (2) high, using the median of the community wealth index scores as reference
Maternal education	Categorised into (1) low (2) high, using the median as reference
Safe water	Categorised into (1) low (2) high, using the median as reference

#### Data analysis

The data were analysed by use of STATA version 15.1^(30)^. At the bivariate level, cross-tabulations were done using Pearson’s chi-squared test for the categorical variables. The Mann–Whitney U and Kruskal–Wallis tests (non-parametric) were used to test for association among the continuous variables with two and more than two independent variables, respectively. These tests were used because the continuous variables were not normally distributed.

Two-level multi-level binary logistic regression analysis was done to investigate the extent to which the individual and community-level factors explained the variation in under- and over-nutrition in the two Nigerian states. This involved 1200 school-aged children and adolescents (level 1) nested within forty communities (level 2). Enumeration areas, as demarcated by the National Population Commission for 2006 population census in Nigeria^(31)^, were used as communities in this study, and a total of forty enumeration areas were selected (twenty in each of the two states). Two separate multi-level analyses identified the individual and contextual factors associated with either under- or over-nutrition. Hence, the nutritional status of the school-aged children and adolescents, which was initially categorised into: (1) thinness; (2) normal and (3) overweight/obese, was recoded to form two different dependent variables which were thinness (categorised as (1) thinness (0) otherwise) and overweight/obesity (categorised as (1) overweight/obese (0) otherwise). Firstly, thinness was retained as ‘thinness’, while normal and overweight/obesity were merged as ‘otherwise’ (1 – Thinness, 0 – otherwise). For the second dependent variable, overweight/obesity were retained as such, while normal and thinness were merged as ‘otherwise’ (1 – overweight/obesity, 0 – otherwise). Six models each (total of twelve models) were fitted in all. The first model (Model 0) was the empty model, and the second model (Model 1) considered only the states (Osun and Gombe States), while the third model (Model 2) incorporated the child characteristics to Model 1. The fourth model (Model 3) incorporated the household/family characteristics into the first model. The community-level factors alone were considered in the fifth model (Model 4), while the sixth model (Model 5) is the full model that incorporated all factors into the multi-level analysis. Ethnicity was not included because of a high variance inflation factor when multi-collinearity diagnostics were done.

The fixed effects were used as the measures of association and expressed as adjusted OR (aOR) with the 95 % CI and the *P* values. The random effects, which measured variations, were intra-class correlation or the variance partition coefficient and the proportional change in variance. Akaike information criteria were used to determine the goodness-of-fit of the models, where a lower value indicated a better fit^(32)^. The independent structure, which is the default for the STATA software, was used in the present study.

## Results

**Fig. 1:**
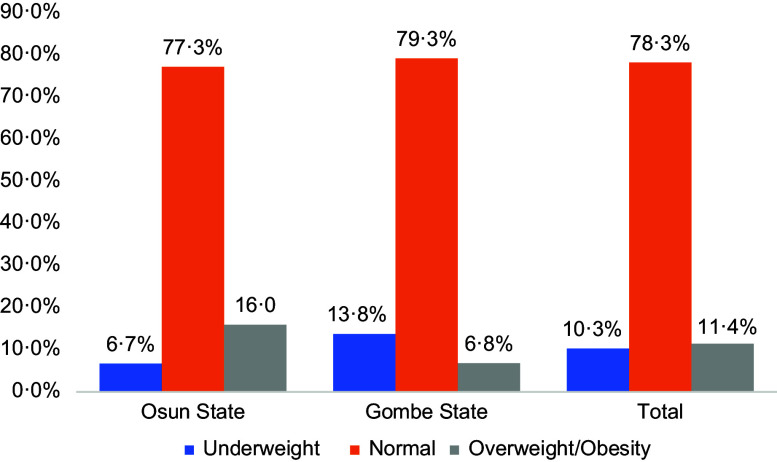
Distribution of the nutritional status among school-aged children and adolescents by state

The prevalence rates of thinness and overweight/obesity were 10·3 % and 11·4 %, respectively, while 21·7 % had one form of malnutrition or the other (Fig. 1). Gombe State has a higher prevalence of thinness (13·8 % *v*. 6·7 %) and lower prevalence of overweight/obesity (6·8 % *v*. 16·0 %). The prevalence rate of malnutrition (under- and over-nutrition) was 22·7 % for Osun State and 20·6 % for Gombe State.

The description of all individual- and community-level factors according to the states is shown in Supplemental Table 1. Table 2 shows that all the child characteristics had a statistically significant association with the nutritional status of the respondents (*P* < 0·05) at the bivariate analysis level, except education (*P* = 0·86), birth order (*P* = 0·06) and pubertal staging (*P* = 0·06). All the household/family factors had a statistically significant association with the nutritional status of the older children (*P* < 0·05), except marital status (*P* = 0·61) and family type (*P* = 0·67). All the community-level factors had a statistically significant association with the nutritional status of the children (*P* < 0·001).

**Table 2:** Association between the individual- and community-level factors and nutritional status at bivariate analysis level

Variables	Nutritional status (%)						Statistics
	Thinness		Normal		Overweight/obesity		
Individual-level factors – child characteristics							
Age of the child (IR)	12·0	7·0	12·0	6·0	10·0	4·0	† *P* < 0·001*
Breastfeeding duration (months) (IR)	18·0	8·0	18·0	9·0	18·0	8·0	† *P* = 0·046*
Sex							
Male	67	11·1	481	80·0	53	8·8	*P* = 0·014*
Female	56	9·3	459	76·6	84	14·0	
Child education							
Currently in-school	116	10·5	866	78·3	124	11·2	*P* = 0·855
Previously in-school	4	7·0	45	78·9	8	14·0	
Never attended school	3	8·1	29	78·4	5	13·5	
Birth weight of the child							
Small (< 2·5 kg)	17	10·2	135	80·8	15	9·0	*P* = 0·003*
Normal (2·5–4 kg)	91	10·9	629	75·6	112	13·5	
Big (> 4 kg)	15	7·5	176	87·6	10	5·0	
Birth place of the child							
Others	32	14·6	167	76·3	20	9·1	*P* = 0·042*
Hospital	91	9·3	773	78·8	117	11·9	
Exclusively breastfed							
No	65	13·1	385	77·6	46	9·3	*P* = 0·006*
Yes	58	8·2	555	78·8	91	12·9	
Immunisation status of child							
Not immunised	6	28·6	13	61·9	2	9·5	*P* = 0·012*
Partially immunised	26	16·5	116	73·4	16	10·1	
Fully immunised	86	8·8	773	79·5	113	11·6	
Not sure	5	10·2	38	77·6	6	12·2	
Birth order of the child							
1	58	9·7	460	77·2	78	13·1	*P* = 0·063
2–4	53	11·2	366	77·5	53	11·2	
> 4	12	9·1	114	86·4	6	4·5	
Screen time (hours) (IR)	2·0	3·0	2·8	3·2	3·0	3·0	† *P* < 0·001*
Physical activity scores (IR)	2·0	1·1	2·3	1·0	2·1	0·8	† *P* < 0·001*
Child’s health							
Very good	39	7·8	391	78·4	69	13·8	*P* < 0·001*
Good	57	9·7	473	80·6	57	9·7	
Not too good	27	23·7	76	66·7	11	9·6	
Pubertal staging							
Stage 1	32	9·7	268	81·2	30	9·1	*P* = 0·061
Stage 2	37	9·2	328	81·2	39	9·7	
Stage 3	35	10·7	238	72·8	54	16·5	
Stage 4	16	13·8	89	76·7	11	9·5	
Stage 5	3	13·0	17	73·9	3	13·0	
Individual-level factors –household/family characteristics							
Household size (IR)	7·0	5·0	6·0	3·0	5·0	2·0	† *P* < 0·001*
Children in the household (IR)	5·0	3·0	4·0	2·0	3·0	1·0	† *P* < 0·001*
Household wealth index							
Poor	61	15·3	294	73·5	45	11·3	*P* < 0·001*
Middle	44	11·0	312	78·0	44	11·0	
Rich	18	4·5	334	83·5	48	12·0	
Marital status							
Single	1	12·5	5	62·5	2	25·0	*P* = 0·606
Married	117	10·4	879	78·1	129	11·5	
Previously married	5	7·5	56	83·6	6	9·0	
Family type							
Monogamous	107	10·1	833	78·3	124	11·7	*P* = 0·667
Polygamous	16	11·8	107	78·7	13	9·6	
Mother’s education							
Less than secondary	61	16·3	283	75·7	30	8·0	*P* < 0·001*
Secondary or more	62	7·5	657	79·5	107	13·0	
Father’s education							
Less than Secondary	35	15·2	170	73·6	26	11·3	*P* = 0·023*
Secondary or more	88	9·1	770	79·5	111	11·5	
Mother’s employment							
Employed	77	8·1	759	79·5	119	12·5	*P* < 0·001*
Unemployed	46	18·8	181	73·9	18	7·3	
Community-level factors							
State							
Gombe	83	13·8	476	79·3	41	6·8	*P* < 0·001*
Osun	40	6·7	464	77·3	96	16·0	
Residence							
Rural	77	12·8	481	80·2	42	7·0	*P* < 0·001*
Urban	46	7·7	459	76·5	95	15·8	
Community wealth index							
Low	79	13·2	477	79·5	44	7·3	*P* < 0·001*
High	44	7·3	463	77·2	93	15·5	
Mother’s education							
Low	85	14·2	472	78·7	43	7·2	*P* < 0·001*
High	38	6·3	468	78·0	94	15·7	
Safe water							
Low	69	14·4	370	77·1	370	77·1	*P* < 0·001*
High	54	7·5	570	79·2	96	13·3	

IR, interquartile range; Screen time, time spent watching television, with phone, computer or computer.

*Statistically significant.

† Kruskal–Wallis test (non-parametric) was used because the variables were not normally distributed.

Table 3 shows the results of the multi-level analyses for thinness (under-nutrition), highlighting the fixed and random effects. The full model shows that household size (aOR 1·10; *P* = 0·001; 95 % CI (1·04, 1·16)) and the uppermost wealth index (rich) (aOR 0·43; *P* = 0·016; 95 % CI (0·22, 0·86)) had a significant positive and inverse associations with thinness, respectively. Concerning the measures of variations for thinness, as shown with the random effects on Table 3, the intra-class correlation for the intercept-only model (i.e. no explanatory variable) was 21·6 %. This implies that 21·6 % of the variation in the odds of thinness was attributable to community-level variables, and this was statistically significant (*P* < 0·001). The Models 1, 2, 3, 4 and 5 account for about 22·1 %, 51·8 %, 41·2 %, 56·7 % and 80·2 % in the odds of under-nutrition across the communities, as explained by the proportional change in variance. The model with the best fit is Model 3, which controlled for State and the Household/family characteristics, with Akaike information criteria of 740·41 compared with 753·3 for the empty model (Model 0).

**Table 3:** Individual and contextual factors associated with thinness among school-aged children and adolescents in two Nigerian states using a two-level multi-level analysis

Variables	Model 0		Model 1		Model 2		Model 3		Model 4		Model 5	
	OR	95 % CI	OR	95 % CI	OR	95 % CI	OR	95 % CI	OR	95 % CI	OR	95 % CI
States												
Gombe ®												
Osun			*0·499	0·25, 0·99	0·521	0·25, 1·09	0·576	0·29, 1·14	0·620	0·33, 1·16	0·696	0·33, 1·48
Individual-level factors – child characteristics												
Age					0·977	0·92, 1·04					0·976	0·91, 1·04
Breastfeeding (months)					1·031	0·99, 1·08					1·029	0·98, 1·08
Gender												
Male ®												
Female					0·933	0·61, 1·43					0·914	0·59, 1·42
Birth weight												
Small (< 2·5 kg) ®												
Normal (2·5–4 kg)					1·248	0·67, 2·31					1·081	0·58, 2·02
Big (> 4 kg)					0·836	0·37, 1·91					0·603	0·26, 1·42
Hospital birth												
No ®												
Yes					0·950	0·55, 1·65					1·284	0·71, 2·31
Child education												
Currently in-school ®												
Previously in school					0·649	0·21, 1·97					0·613	0·19, 1·97
Never in school					0·341	0·09, 1·29					0·379	0·10, 1·43
Immunisation												
Not immunised ®												
Partially immunised					0·786	0·23, 2·71					1·063	0·26, 4·28
Fully immunised					0·566	0·16, 1·97					0·958	0·23, 3·99
Not sure					0·559	0·12, 2·58					0·508	0·09, 2·78
Birth order												
1 ®												
2–4					1·017	0·65, 1·59					1·022	0·64, 1·63
> 4					0·737	0·36, 1·53					0·537	0·22, 1·32
Exclusive breastfeeding												
No ®												
Yes					0·828	0·49, 1·39					0·895	0·52, 1·54
Screen time												
< 2 h ®												
≥ 2 h					0·677	0·43, 1·08					0·805	0·49, 1·32
Physical activity scores					1·072	0·80, 1·45					0·963	0·71, 1·30
Child’s health												
Very good ®												
Good					1·079	0·64, 1·83					0·951	0·55, 1·65
Not too good					1·458	0·63, 3·38					1·127	0·46, 2·76
Puberty stage												
1 ®												
2					1·193	0·67, 2·11					1·282	0·71, 2·30
3					1·179	0·63, 2·20					1·463	0·77, 2·78
4					1·937	0·87, 4·32					1·506	0·65, 3·48
5					2·602	0·70, 9·68					2·350	0·60, 9·19
Individual-level factors – household/family characteristics												
Household size							**1·076	1·02, 1·13			***1·097	1·04, 1·16
Children in the household							0·936	0·85, 1·03			0·972	0·86, 1·10
Wealth index												
Poor ®												
Middle							0·827	0·51, 1·33			0·921	0·55, 1·53
Rich							***0·350	0·19, 0·66			*0·432	0·22, 1·86
Marital status												
Single ®												
Married							0·596	0·06, 5·56			0·449	0·05, 4·32
Previously married							0·408	0·04, 4·58			0·351	0·03, 4·25
Family type												
Monogamous ®												
Polygamous							0·519	0·25, 1·07			0·452	0·20, 1·02
Maternal education												
< secondary ®												
Secondary or more							0·775	0·45, 1·32			0·796	0·44, 1·44
Father’s education												
< Secondary ®												
Secondary or more							1·042	0·60, 1·80			1·204	0·67, 2·17
Mother’s employment												
Employed ®												
Unemployed							1·111	0·66, 1·87			1·397	0·81, 2·41
Community-level factors												
Residence												
Rural ®												
Urban									1·873	0·63, 5·55	1·677	0·62, 4·53
Community wealth index												
Low ®												
High									0·553	0·28, 1·07	0·592	0·32 1·10
Maternal education												
Low												
High ®									*0·332	0·12, 1·92	0·459	0·18, 1·19
Safe water												
Low ®												
High									0·637	0·33, 1·21	0·733	0·41, 1·32
Random effects												
Community levelVariance (se)	***0·905	0·34	***0·705	0·29	***0·436	0·23	***0·532	0·25	***0·392	0·21	0·179	0·19
VPC = ICC (%)	21·57 %		17·65 %		11·69 %		13·91 %		10·64 %		5·16 %	
Explained variation (ie PCV in %)	Reference		22·10 %		51·82 %		41·22 %		56·69 %		80·22 %	
Log likelihood	−374·65		−372·85		−356·26		−357·20		−367·15		−337·18	
Model fit statistics (AIC)	753·30		751·70		762·51		740·41		748·31		752·35	

R, reference value; Screen time, time spent watching television, with phone, computer or computer games; VPC, variance partition coefficient; ICC, intra-class correlation; PCV, proportional change in variance; AIC, Akaike information criteria.

Statistically significant: *P < 0·005; **P < 0·010; ***P < 0·001.

Table 4 shows the results of the multi-level analysis for overweight/obesity (over-nutrition). Age (aOR 0·86; *P* < 0·001; 95 % CI (1·26, 8·70)), exclusive breastfeeding (aOR 0·46; *P* = 0·010; 95 % CI (0·25, 0·83)), physical activity (aOR 0·55; *P* = 0·001; 95 % CI (0·39, 0·78)) and the rich wealth index (aOR 0·47; *P* = 0·018; 95 % CI (0·25, 0·88)) had an inverse relationship with overweight/obesity, while residing in Osun State (aOR 3·32; *P* = 0·015; 95 % CI (1·26, 1·70)), female gender (aOR 1·73; *P* = 0·015; 95 % CI (1·11, 2·69)) and screening time > 2 h/d (aOR 2·33; *P* = 0·005; 95 % CI (1·29, 4·19)) were positively associated with overweight/obesity. The empty model (Model 0) shows a statistically significant variation in the odds of childhood overweight/obesity across the communities (*P* < 0·001). As indicated by the intra-class correlation, 28·5 % of the variance in the odds of overweight/obesity among the children could be ascribed to community-level factors. The Models 1, 2, 3, 4 and 5 account for about 23·4 %, 35·3 %, 18·2 %, 48·1 % and 39·2 % in the odds of over-nutrition across the communities, as explained by the proportional change in variance. The statistically significant variation across communities persisted even after controlling for all the variables (*P* < 0·001). Model 2, which controlled for the state and child characteristics, had the best fit with Akaike information criteria of 752·76. (Table 4).

**Table 4:** Individual and contextual factors associated with over-nutrition among school-aged children and adolescents in two Nigerian states using a two-level multi-level analysis

Variables	Model 0		Model 1		Model 2		Model 3		Model 4		Model 5	
	OR	95 % CI	OR	95 % CI	OR	95 % CI	OR	95 % CI	OR	95 % CI	OR	95 % CI
States												
Gombe ®												
Osun			*2·499	1·16, 5·38	**3·930	1·61, 9·57	2·138	0·93, 4·91	**2·543	1·23, 5·28	*3·317	1·26, 8·70
Individual-level factors – child characteristics												
Age					***0·864	0·81, 0·93					***0·861	0·80, 0·92
Breastfeeding (months)					0·986	0·95, 1·03					0·990	0·95, 1·03
Gender												
Male ®												
Female					*1·748	1·13, 2·70					*1·731	1·11, 2·69
Birth weight												
Small (< 2·5 kg) ®												
Normal (2·5–4 kg)					1·143	0·57, 2·29					1·180	0·57, 2·45
Big (> 4 kg)					0·491	0·19, 1·29					0·552	0·21, 1·48
Hospital birth												
No ®												
Yes					1·138	0·61, 2·14					0·993	0·51, 1·92
Child education												
Currently in-school ®												
Previously in school					2·057	0·82, 5·16					2·023	0·79, 5·16
Never in school					1·309	0·35, 4·87					1·177	0·30, 4·57
Immunisation												
Not immunised ®												
Partially immunised					1·035	0·17, 6·20					1·143	0·19, 6·75
Fully immunised					0·682	0·12, 3·88					0·752	0·13, 4·23
Not sure					1·171	0·16, 8·47					1·553	0·22, 11·18
Birth order												
1 ®												
2–4					1·068	0·69, 1·64					1·129	0·72, 1·78
> 4					0·562	0·21, 1·48					0·615	0·20, 1·91
Exclusive BF												
No ®												
Yes					*0·514	0·29, 0·91					**0·455	0·25, 0·83
Screen time												
< 2 h ®												
≥ 2 h					**2·159	1·23, 3·78					**2·329	1·29, 4·19
Physical activity scores					***0·551	0·39, 0·77					***0·550	0·39, 0·78
Child’s health												
Very good ®												
Good					0·725	0·42, 1·25					0·712	0·41, 1·24
Not too good					1·822	0·65, 5·08					1·924	0·65, 5·66
Puberty stage												
1 ®												
2					1·073	0·61, 1·89					1·090	0·61, 1·93
3					1·272	0·67, 2·42					1·283	0·67, 2·46
4					1·028	0·43, 2·46					1·082	0·44, 2·65
5					0·560	0·06, 4·96					0·601	0·07, 5·41
Individual-level factors – household/family characteristics												
Household size							1·003	0·93, 1·08			0·997	0·91, 1·09
Children in the household							0·937	0·82, 1·07			0·991	0·85, 1·16
Wealth index												
Poor ®												
Middle							0·760	0·46, 6·15			0·686	0·39, 1·20
Rich							0·589	0·34, 5·05			*0·472	0·25, 0·88
Marital status												
Single (R)												
Married							0·933	0·14, 6·15			1·108	0·13, 9·63
Previously married							0·644	0·08, 5·05			0·677	0·07, 6·98
Family type												
Monogamous (R)												
Polygamous							1·440	0·67, 3·12			1·646	0·69, 3·90
Maternal education												
Less than secondary (R)												
Secondary or more							1·730	0·93, 3·23			2·052	0·98, 4·32
Father’s education												
Less than Secondary (R)												
Secondary or more							0·617	0·33, 1·15			0·622	0·32, 1·21
Mother’s employment												
Employed (R)												
Unemployed							0·923	0·49, 1·75			0·731	0·36, 1·47
Community-level factors												
Residence												
Rural (R)												
Urban									2·275	0·55, 9·48	1·195	0·24, 5·89
Community wealth index												
Low (R)												
High									1·696	0·78, 3·71	1·565	0·65, 3·76
Maternal education												
Low												
High (R)									0·936	0·26, 3·40	1·612	0·37, 7·05
Safe water												
Low (R)												
High									0·698	0·31, 1·58	0·731	0·30, 1·80
Random effects												
Community-level variance (se)	***1·311	0·48	***1·005	0·39	***0·848	0·37	***1·073	0·43	***0·681	0·30	***0·796	0·36
VPC = ICC (%)	28·50 %		23·40 %		20·49 %		24·60 %		17·14 %		19·48 %	
Explained variation (i.e. PCV in %)	Reference		23·34 %		35·32 %		18·15 %		48·05 %		39·21 %	
Log likelihood	−391·56		−389·07		−351·01		−384·50		−385·41		−343·87	
Model fit statistics (AIC)	787·11		784·15		752·03		795·00		784·82		765·73	

R, reference value; Screen time, time spent watching television, with phone, computer or computer games; VPC, variance partition coefficient; ICC, intra-class correlation; PCV, proportional change in variance; AIC, Akaike information criteria.

Statistically significant: *P < 0·005; **P < 0·010; ***P < 0·001.

## Discussion

This study, to the best our knowledge, is the first attempt to simultaneously consider the influence of individual- and community-level factors as predictors of under- and over-nutrition among school-aged and adolescents in Nigeria. Most previous studies have focussed on various indicators of under-nutrition among categories of under-five children. It is also the first study that used multi-level analysis to identify the predictors of overweight/obesity among any group of children or adolescents in Nigeria. Furthermore, the present study controlled for a wide range of independent variables, making it one of the most comprehensive studies on the determinants of nutritional status of any category of children and/or adolescents in Nigeria.

The ecological systems theory has underscored the importance of not only the compositional factors (i.e. the individual-level factors), but also the contextual factors (i.e. community-level factors) in trying to understand a complex and highly important process like child nutrition^(6,7)^. The multi-level analysis that was done in this study therefore helped to account for the variations in under- and over-nutrition of older children across the different contextual units, as well as to identify the compositional and contextual factors that were associated with under- and over-nutrition among the older children in the two Nigerian States.

A major finding of this study is the importance of community variation and community-level factors in the prevalence of thinness and overweight/obesity among children 6–19 years old in the two Nigerian states. As indicated by the intra-class correlation of the intercept-only model (i.e. no explanatory variable incorporated), about 22 % and 29 % of the variance in the odds for thinness and overweight/obesity among the children could be ascribed to community-level factors, respectively. Apart from the full model that included all explanatory variables, the model which consisted of community-level factors only (these factors include the state, residence, wealth index, maternal education and safe water), accounted for the highest variation observed for children that were thin (57 %) and overweight/obese (48 %). This suggests that school-aged children and adolescents from the same communities are influenced by common factors, and hence the potential for community-level interventions.

The present study found the odds for thinness increased by 10 % for every unit increase in household size. Not many studies among children have explored the relationship between under-nutrition and household size, but some studies have however looked at other variables that could serve as proxy for the household size. For example, Nnebue *et al*.^(33)^ found a statistically significant relationship between under-nutrition and the number of siblings a child has. This finding further underscores the need for increased efforts in promoting family planning in Nigeria, because a larger number of children may put a household at higher risk of poverty and hence of under-nutrition. The household wealth index was also significantly associated with thinness, such that those in the ‘rich’ category had 57 % lesser odds of being thinner than those in the ‘poor’ category. Furthermore, wealth index had a statistically significant association with household size, such that those from richer households significantly had a lower household size. Hence, large family size, which increased the likelihood of thinness, may be a proxy for poverty. The relationship between poverty and under-nutrition, especially in low and middle-income countries has been well established^(34)^. Therefore, effective interventions for thinness (under-nutrition) in these two states and Nigeria as a whole may be interventions against poverty.

The present study found that for every unit increase in age, the odds of being overweight/obese reduced by 14 %. The association between age and overweight/obesity may not be unconnected with the pubertal staging of the respondents. Although the pubertal stage was not significantly associated with overweight/obesity, it had a strong statistically significant association with age (*P* < 0·001). Females were also found to have two times higher odds of being overweight/obese than males. The relationship between overweight/obesity, age and sex of school-aged children and adolescents has been similarly reported by other studies within and outside Nigeria^(35–37)^. Screen time (i.e. time spent with television, computers, video games and phones) of 2 h or more daily had two times higher odds of being overweight/obese, while the odds of being overweight/obese reduced by 45 % with a unit increase in physical activity. This finding is in line with previous studies linking overweight/obesity to reducing physical activity and increasing sedentary lifestyle, of which screen time plays a major role^(14,15,38,39)^.

Increasing screen time has also been associated with higher consumption of snacks, which also has been reported to significantly increase the likelihood of overweight/obesity in children^(40)^. Exclusive breastfeeding was found to significantly reduce the odds of being overweight/obesity by as much as 54 % in line with the results of previous studies^(41–43)^. This finding is important as it underscores the importance of exclusive breastfeeding in reducing, not only childhood under-nutrition and mortality^(21,44)^, but also overweight/obesity. An interesting finding was that being part of a rich household reduced the odds of overweight/obesity by 53 %. This is different from what previous researchers in Nigeria have reported^(37,39)^, but similar to the finding in the high-income countries^(45)^. This may be due to community-level factors which were probably not assessed in this study, since 30 % of the variance in the odds for overweight/obesity in this study is attributable to community-level factors. Community-level factors such as availability, accessibility and proximity to fast-food shops and recreational facilities or programmes in communities have been shown to influence the nutritional status of children and adolescents^(46–48)^. Another plausible reason for this is that, by reason of exposure, the richest households in Nigeria are already adopting the lifestyle and values of the rich in developed countries where emphasis is placed on healthy food, exercises and a slim figure.

The school-aged children and adolescents living in Osun State had three times higher odds of being overweight/obese than those from Gombe State, and this may be a reflection of the higher socio-economic status and urbanisation of Osun State and the southwestern part of Nigeria compared with the northeastern part of the country where Gombe State is located^(49)^. In the present study, although the wealth index was not significantly different between the two states, Osun state did significantly better for almost all other indices of better socio-economic status than Gombe state, including household size, number of children in the family, family type, mother’s education, father’s education and mother’s employment.

Comparing the findings of the present study with those from previous multi-level analyses in Nigeria is challenging. Firstly, the previous studies were undertaken for under-five children, and they also focussed on under-nutrition alone. Furthermore, no previous research effort has used multi-level modelling to understand the determinants of overweight/obesity among any group of children/adolescents in Nigeria^(20,21,24)^. Additionally, the reference values used in the present study are different from those used by other previous studies. Previous studies used height-for-age (for stunting)^(20,21)^, weight-for-age (for underweight)^(24)^ and weight-for-height (for wasting)^(24)^ reference values, which are all indicators of under-nutrition. The present study used the BMI-for-age reference values, which has the advantage of measuring both under-nutrition (measured as thinness) and over-nutrition (measured as overweight and obesity)^(28)^.

A limitation of this study is that the findings of this study may not be generalisable to all of Nigeria, because only two out of thirty-six states were involved in the study. Another limitation is that, as to date, there was no data about the contextual determinants of overweight/obesity among older children in Nigeria using multi-level analysis, hence making the comparison of the findings of the present study and others challenging. The cross-sectional nature of the study also makes it impossible to establish causality.

### Policy implications

The findings of this study have some important policy implications. The present study observed that the community-level factors contributed significantly to the odds of under- and over-nutrition, indicating the need to explore community-based nutritional interventions for school-aged children and adolescents. There is also a need to review current interventions to assess whether/how they could be scaled-up and targeted at reducing socio-economic in-equalities, and especially poverty among households in the study location. The food systems approach of the Food and Agriculture Organization to create an enabling environment for improved nutrition^(50)^ can guide governance for improved nutrition, evidence-based policies and programmes and financial investment to facilitate changes in food systems. The findings of the present study underscore the importance of reduced physical activity and prolonged screen time in increasing the odds for overweight/obesity among the school-aged children and adolescents. There is a need, therefore, for the development of recreational, sports or games centres and programmes for children and adolescents in different communities that will increase engagement in physical activity and reduce screen time among them.

## Conclusion

This study showed that Nigeria faces the challenge of a double burden of malnutrition among its school-aged and adolescents (6–19 years), with over a fifth experiencing either under-nutrition or over-nutrition. The study showed thinness and overweight/obesity among school-aged children and adolescents were strongly influenced by their communities, individual-level factors and their residence. Predictors of thinness in this study were household size and household wealth index. Overweight/obesity was significantly associated with the age, sex, exclusive breastfeeding, physical activity and household wealth index. Policymakers and stakeholders should therefore plan community-based educational programs to address, especially, socio-economic status, physical activity patterns among the children and the control of family/household size in the two Nigerian states.
