# Individual and contextual factors associated with under- and over-nutrition among school-aged children and adolescents in two Nigerian states: a multi-level analysis – CORRIGENDUM

**DOI:** 10.1017/S1368980022001082

**Published:** 2022-08

**Authors:** Adeleye Abiodun Adeomi, Adesegun Fatusi, Kerstin Klipstein-Grobusch

The authors regret the inclusion of an error in the above article.

In the funding statement, the grant numbers were originally given as ‘B 8606.RO2’ and ‘54100029’.

The correct grant numbers are ‘G-19-57145’ and ‘54100113’.

The article has been corrected.
